# Identification of candidate metabolite biomarkers for metabolic syndrome and its five components in population-based human cohorts

**DOI:** 10.1186/s12933-023-01862-z

**Published:** 2023-06-16

**Authors:** Mengya Shi, Siyu Han, Kristin Klier, Gisela Fobo, Corinna Montrone, Shixiang Yu, Makoto Harada, Ann-Kristin Henning, Nele Friedrich, Martin Bahls, Marcus Dörr, Matthias Nauck, Henry Völzke, Georg Homuth, Hans J. Grabe, Cornelia Prehn, Jerzy Adamski, Karsten Suhre, Wolfgang Rathmann, Andreas Ruepp, Johannes Hertel, Annette Peters, Rui Wang-Sattler

**Affiliations:** 1 TUM School of Medicine, Technical University of Munich (TUM), Munich, Germany; 2 Institute of Translational Genomics, Helmholtz Zentrum München, German Research Center for Environmental Health, Neuherberg, Germany; 3 German Center for Diabetes Research (DZD), Partner Neuherberg, Munich-Neuherberg, Germany; 4 Department of Psychiatry and Psychotherapy, University Medicine Greifswald, Greifswald, Germany; 5 Institute of Experimental Genetics, Helmholtz Zentrum München, German Research Center for Environmental Health, Neuherberg, Germany; 6 Institute of Clinical Chemistry and Laboratory Medicine, University Medicine Greifswald, Greifswald, Germany; 7 German Centre for Cardiovascular Research (DZHK), Partner Site Greifswald, Greifswald, Germany; 8 Department of Internal Medicine B, University Medicine Greifswald, Greifswald, Germany; 9 Institute for Community Medicine, University Medicine Greifswald, Greifswald, Germany; 10 German Centre for Diabetes Research (DZD), Partner Greifswald, Neuherberg, Germany; 11 Interfaculty Institute for Genetics and Functional Genomics, University Medicine Greifswald, Greifswald, Germany; 12 Metabolomics and Proteomics Core, Helmholtz Zentrum München, German Research Center for Environmental Health, Neuherberg, Germany; 13 Department of Biochemistry, Yong Loo Lin School of Medicine, National University of Singapore, Singapore, Singapore; 14 Institute of Biochemistry, Faculty of Medicine, University of Ljubljana, Ljubljana, Slovenia; 15 Department of Physiology and Biophysics, Weill Cornell Medicine—Qatar, Education City—Qatar Foundation, Doha, Qatar; 16 German Center for Diabetes Research (DZD), Partner Düsseldorf, Neuherberg, Germany; 17 Institute of Biometrics and Epidemiology, German Diabetes Center, Leibniz Center for Diabetes Research at Heinrich-Heine-University Düsseldorf, Düsseldorf, Germany; 18 German Center for Neurodegenerative Diseases (DZNE), Greifswald, Germany; 19 Institute of Epidemiology, Helmholtz Zentrum München, German Research Center for Environmental Health, Neuherberg, Germany; 20 Institute for Medical Information Processing, Biometry, and Epidemiology, Pettenkofer School of Public Health, Ludwig Maximilian University of Munich (LMU), Munich, Germany; 21 Munich Heart Alliance, German Center for Cardiovascular Health (DZHK E.V., Partner-Site Munich), Munich, Germany

**Keywords:** Metabolic syndrome, Obesity, Cardiovascular disease, Hypertension, Hyperglycemia, Metabolomics, Amino acids, BCAAs, Phosphatidylcholines, Lysophosphatidylcholines

## Abstract

**Background:**

Metabolic Syndrome (MetS) is characterized by risk factors such as abdominal obesity, hypertriglyceridemia, low high-density lipoprotein cholesterol (HDL-C), hypertension, and hyperglycemia, which contribute to the development of cardiovascular disease and type 2 diabetes. Here, we aim to identify candidate metabolite biomarkers of MetS and its associated risk factors to better understand the complex interplay of underlying signaling pathways.

**Methods:**

We quantified serum samples of the KORA F4 study participants (N = 2815) and analyzed 121 metabolites. Multiple regression models adjusted for clinical and lifestyle covariates were used to identify metabolites that were Bonferroni significantly associated with MetS. These findings were replicated in the SHIP-TREND-0 study (N = 988) and further analyzed for the association of replicated metabolites with the five components of MetS. Database-driven networks of the identified metabolites and their interacting enzymes were also constructed.

**Results:**

We identified and replicated 56 MetS-specific metabolites: 13 were positively associated (e.g., Val, Leu/Ile, Phe, and Tyr), and 43 were negatively associated (e.g., Gly, Ser, and 40 lipids). Moreover, the majority (89%) and minority (23%) of MetS-specific metabolites were associated with low HDL-C and hypertension, respectively. One lipid, lysoPC a C18:2, was negatively associated with MetS and all of its five components, indicating that individuals with MetS and each of the risk factors had lower concentrations of lysoPC a C18:2 compared to corresponding controls. Our metabolic networks elucidated these observations by revealing impaired catabolism of branched-chain and aromatic amino acids, as well as accelerated Gly catabolism.

**Conclusion:**

Our identified candidate metabolite biomarkers are associated with the pathophysiology of MetS and its risk factors. They could facilitate the development of therapeutic strategies to prevent type 2 diabetes and cardiovascular disease. For instance, elevated levels of lysoPC a C18:2 may protect MetS and its five risk components. More in-depth studies are necessary to determine the mechanism of key metabolites in the MetS pathophysiology.

**Supplementary Information:**

The online version contains supplementary material available at 10.1186/s12933-023-01862-z.

## Background

Metabolic Syndrome (MetS) is diagnosed by the presence of at least three out of five risk factors: abdominal obesity, hypertriglyceridemia, reduced high-density lipoprotein cholesterol (HDL-C), hypertension, and hyperglycemia [1]. This diagnosis carries a two-fold risk of developing cardiovascular disease (CVD) within 5–10 years and a five-fold greater risk of developing type 2 diabetes (T2D) [1]. Furthermore, individuals with MetS have a higher rate of developing serious complications from severe acute respiratory syndrome coronavirus 2 (SARS-CoV-2) infection [2]. Although global data on MetS is lacking, a study conducted in 2018 estimated that more than one billion people worldwide suffer from MetS [3], making it a significant public health issue due to its association with serious chronic complications such as CVD and T2D.

The etiology of MetS is attributed to multiple interacting factors such as genetic susceptibility, epigenetic factors, and environmental influences, as well as the five metabolic components [4]. Obesity, for instance, is largely influenced by socioeconomic and dietary factors, with excessive caloric intake possibly leading to inflammatory conditions and impaired energy metabolism [5]. Dyslipidemia, characterized by the imbalance of lipids such as raised triglycerides and lower HDL–C, is considered to be a risk factor for the development of atherosclerotic CVD [1]. Excessive food intake and abnormal lipid metabolism have been reported to be associated with the development of atherogenic dyslipidemia [6]. Hypertension, another significant component of MetS, arises from oxidative stress, endothelial dysfunction, and increased inflammatory mediators, posing a major risk for CVDs, including stroke [4]. In addition, hyperglycemia and insulin resistance exacerbate low-grade inflammation, enhancing the risk of CVDs [7]. Therefore, besides lifestyle modifications and psychological management, understanding the pathophysiology of MetS is vital for developing pharmaceutical interventions for personalized medicine [2].

Technological advancements enable the quantification and analysis of multi-omics data such as genomic, proteomic, and metabolomic data in clinical studies and human cohorts [8]. The comprehensive analysis of molecules (genes, proteins, and metabolites) may contribute to a systematic understanding of biological processes and to the development of personalized therapies [9]. Metabolome refers to the complete set of small molecule metabolites, which are the intermediates or end products of metabolism, and reflect the metabolic status of an individual or a population. By applying advanced analytical and statistical methods, metabolomics has the potential to reveal novel biomarkers that can improve the diagnosis, prognosis, and risk assessment of MetS and its related disorders [10, 11].

In particular, the utilization of the targeted metabolomics approach in previous studies provides a robust foundation for our current research, enabling comparisons and the identification of metabolites and potential biomarkers associated with the studied population. For instance, in the population-based KORA (Cooperative Health Research in the Region of Augsburg) study, targeted metabolomics has been intensively used to explore the association between metabolites and age, gender, smoking, alcohol intake, pre-diabetes, and T2D, as well as chronic kidney disease [12–18]. Similarly, in the SHIP (Health Study of Pomerania)-TREND-0 study, targeted metabolomics has been used to investigate the metabolites associated with the use of oral contraceptives [19].

Several metabolomic studies on MetS have employed various approaches, such as untargeted, targeted metabolomic, and lipidomic, revealing novel biomarkers and providing new insights into metabolic alterations in MetS [20–23]. However, previous studies have often utilized relatively smaller sample sizes (e.g., 30 individuals with MetS), or only 100 men [20, 22, 23]. To date, there remains a gap in research focusing on large population-based human cohorts. Therefore, to better understand the underlying mechanisms of MetS, it is necessary to conduct further research utilizing metabolomic approaches in larger sample sizes.

The present study, based on a targeted metabolomics approach, aims to identify MetS-associated metabolites in a population-based human cohort, namely the KORA F4 study [24], and replicate the findings in the SHIP-TREND-0 study [25]. The secondary objective is to investigate the association between the replicated MetS-associated metabolites and the five components of MetS, as well as to construct interaction networks among identified metabolites, enzymes, and biochemical processes to better understand the underlying mechanisms of MetS.

## Methods

### Description of human cohorts: discovery and replication studies

**Fig. 1:**
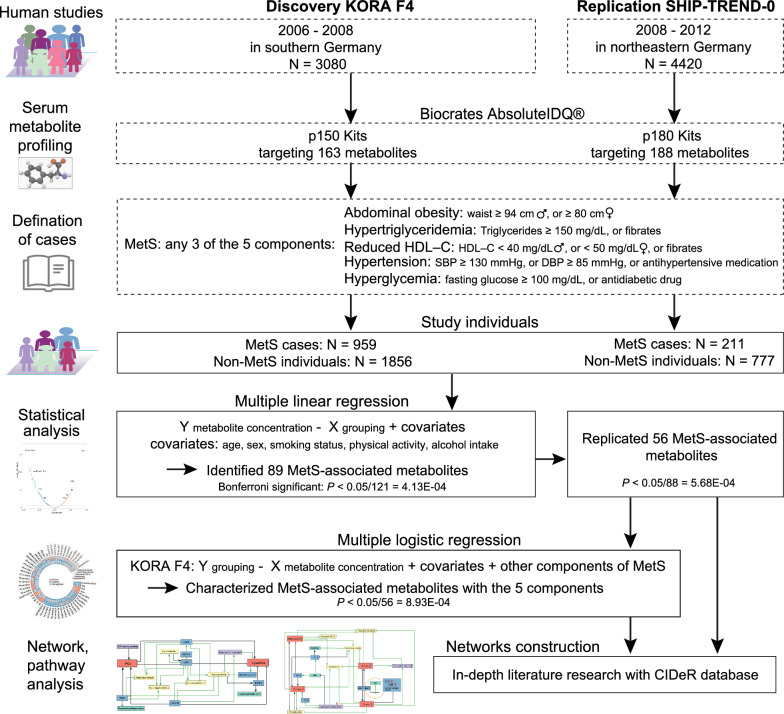
Population description and study design. Abbreviations: SBP, systolic blood pressure; DBP, diastolic blood pressure

*Discovery study* The discovery study utilized data from the KORA F4, a follow-up to the KORA baseline survey number 4 (KORA S4) [24, 26]. The KORA F4 study, conducted between 2006 and 2008, examined 3080 individuals (aged 32–81 years). The clinical variables, demographics, and laboratory measurements of KORA F4 have been described in detail in our previous reports [27, 28]. Only participants with metabolite measurements and clinical variables for MetS were included. We excluded: (1) missing data on phenotypes and metabolites (N = 51); (2) the presence of metabolites’ extreme outliers detected by data points outside of the mean ± 5 × standard deviation range (N = 201); (3) non-fasting samples (N = 7); (4) and missing diagnosis of MetS (N = 6). In total, we included 2815 individuals in this discovery study (Fig. 1).

*Replication study* The replication study was based on the SHIP-TREND-0 study, a population-based study in northeastern Germany, which recruited 4,420 participants between 2008 and 2012 [25, 29, 30]. Details of the measurements performed in the SHIP-TREND-0 study were published [19, 25, 31]. The replication study included 988 individuals with data on MetS and metabolite measurements (Fig. 1).

KORA and SHIP studies were approved by the Ethics Committees of the Bavarian Medical Association in Munich, and the Institutional Review Board of the University of Greifswald, Germany, respectively. All study participants provided written informed consent.

### The definition of MetS

The definition of MetS in KORA F4 and SHIP-TREND-0 studies was based on a joint scientific statement published in 2009 [1, 9]. MetS is diagnosed by the presence of any three of the following five components: (1) abdominal obesity with waist circumferences that measure ≥ 94 cm for men, or ≥ 80 cm for women; (2) hypertriglycerides with fasting serum triglycerides ≥ 150 mg/dL, or drug treatment for elevated triglycerides (fibrates); (3) low serum HDL–C < 40 mg/dL in men, or < 50 mg/dL in women, or drug treatment for reduced HDL–C (fibrates); (4) hypertension with systolic blood pressure ≥ 130 mmHg, or diastolic blood pressure ≥ 85 mmHg, or antihypertensive medication treatment; (5) hyperglycemia with fasting serum glucose level ≥ 100 mg/dL or antidiabetic drug treatment. According to the MetS diagnostic criteria, the KORA F4 and SHIP-TREND-0 participants were divided into MetS and non-MetS groups (Fig. 1).

### Metabolite quantification and normalization

Serum samples from the KORA F4 study were measured with the Absolute*IDQ*™ p150 kit (BIOCRATES Life Sciences AG, Innsbruck, Austria) for the quantification of 163 metabolites [12]. Specifically, samples were randomly distributed on 38 kit plates, each plate also including three quality control (QC) samples provided by the manufacturer and one zero sample (PBS) in addition to the individual samples [18, 32]. Among the quantified metabolites, only those that met all of the following three criteria were used: (1) missing values < 10%; (2) median relative standard deviations** (**RSD, also called coefficient of variation (CV)) of three QC samples < 25%; (3) 50% of measured sample values equal or above the limits of detection (LOD). In total, 121 metabolites that met QC included 14 amino acids, 1 monosaccharide, 18 acylcarnitines, 67 phosphatidylcholines (PCs), 9 lysoPCs (LPCs), and 12 sphingomyelins (Additional file 1: Table S1).

To minimize the technical variations that metabolomics data inevitably contain, metabolite concentrations were adjusted by a non-parametric method TIGER, which is based on an adaptable ensemble learning architecture [32]. In addition, to ensure comparability between different metabolites, their values were natural-log transformed and standardized to have a mean value of 0 and a standard deviation of 1.

In the SHIP-TREND-0 study, metabolic profiling was measured using the Absolute*IDQ*™ p180 kit (BIOCRATES Life Sciences AG, Innsbruck, Austria). Metabolites from the SHIP-TREND-0 that passed quality control were used for replication. A detailed description of the metabolite measurements, as well as pre-processing, has been provided in published papers [19, 33]. The metabolomics measurements in the SHIP-TREND-0 study were performed for a subset of participants without self-reported diabetes. Nonetheless, the study included participants with prediabetes, along with a sufficient number of individuals making the analysis robust and generalizable (Fig. 1).

### Statistical analysis

We first used a multiple linear regression model to identify MetS-associated metabolites with the metabolite concentration values as dependent variable and the grouping variable as independent variable. To include potential confounders, we adjusted for age, sex and smoking status, physical activity, and alcohol intake. In the KORA study, we used the multiple linear regression model: Y _metabolite concentration_ ~ X _grouping (non-MetS = 0, prevalent MetS = 1)_ + age _(years)_ + sex _(female = 0, male = 1)_ + smoking status _(non-smoker = 0, former smoker = 1, and current smoker = 2)_ + physical activity _(inactive = 0, active =1; regular exercise per week ≥ 1 h is considered active, irregular equal or less than 1 h is considered inactive)_ + alcohol intake _(g/day)._ Each metabolite was analyzed separately. To account for multiple testing for the 121 utilized metabolites, a Bonferroni cutoff was applied and only metabolites with *P*-value < 0.05/121 = 4.13E-04 were considered to be statistically significantly associated with MetS in the KORA F4 study (Fig. 1).

To replicate MetS-associated metabolites, in the SHIP-TREND-0 study, we used similar multiple linear regression model: Y _metabolite concentration_ ~ X _grouping (non-MetS = 0, prevalent MetS = 1)_ + age _(years)_ + sex _(female = 2, male = 1)_ + smoking status _(non-smoker = 0, former smoker = 1, and current smoker = 2)_ + physical activity _(inactive = 0, active =1)_ + alcohol intake _(g/day)_. Each metabolite was analyzed separately. A Bonferroni cutoff was applied for multiple testing corrections and only metabolites with a *P*-value < 0.05/88 = 5.68E-04 were considered significant in the SHIP-TREND-0 study.

A multiple logistic regression analysis was further employed to evaluate the association between identified and replicated MetS-associated metabolites and each of the five MetS components in the KORA F4 study. In the multiple logistic regression model, the grouping variable of each component of MetS was defined as outcome and metabolite concentration values as explanatory variables: Y _grouping (non-component 1 = 0, prevalent component 1 = 1)_ ~ X _metabolite concentration_ + age + sex + smoking status + physical activity + alcohol intake + other four components. For each of the components of MetS, each metabolite was assessed individually. For multiple testing correction, a Bonferroni cutoff was applied and only metabolites with *P*-value < 0.05/56 = 8.93E-04 were considered to be statistically significantly associated with the component of MetS in the KORA F4 study (Fig. 1).

All analyses were carried out using R, version 4.1.2, and STATA/MP 17.

### Construction of metabolic networks

CIDeR is a manually curated multifactorial database that integrates interactions between heterogeneous factors associated with human diseases [34]. Data in CIDeR has been sourced from research articles and reviews, the annotations for all interactions were manually annotated by experienced biocurators.

Based on the CIDeR database, in-depth literature research was conducted on the identified metabolites to examine the underlying mechanisms, linked biological processes, clinical phenotypes, and enzymes (protein and encoding gene) that are specifically associated with the five components of MetS.

## Results

### Characteristics of discovery and replication study participants

Table 1 shows the characteristics of the KORA F4 study participants (N = 2815), which included 959 participants with MetS and 1,856 individuals without MetS (Fig. 1). Significant differences in age and sex were observed between the two groups. Participants with MetS were notably older and included a higher proportion of males. Consistent with the diagnostic criteria, individuals with MetS had higher BMI and waist circumferences. Regarding blood measurements, the levels of triglycerides, HDL–C, blood pressure, fasting glucose, and HbA1c were worse in the MetS group than in the non-MetS group. As can be seen from Table 1, smoking status differed significantly between the two groups, with a higher proportion of former smokers in the MetS group. Furthermore, the non-MetS group had a larger proportion of physically active participants, while alcohol intake did not demonstrate statistical differences between the groups.

**Table 1:** Characteristics of discovery and replication studies^a^

	Discovery KORA F4			Replication SHIP-TREND-0		
	MetS	Non-MetS	*P-*value	MetS	Non-MetS	*P-*value
N	959	1856		211	777	
Age (years)	63.00 [54.00, 71.00]	51.00 [42.00, 63.00]	< 0.001	55.94 [51.70,60.19]	48.47 [34.52,62.42]	< 0.001
Sex (% of male)	565 (58.9)	770 (41.5)	< 0.001	118 (0.56)	316 (0.41)	< 0.001
BMI (kg/m^2^)	29.87 [27.46, 32.77]	25.41 [23.16, 28.24]	< 0.001	31.13 [26.88,35.37]	26.31 [22.20,30.41]	< 0.001
Waist circumference (cm)	102.40 [95.90, 110.27]	88.40 [79.40, 95.90]	< 0.001	99.53 [99.25,99.81]	84.87 [84.51,85.22]	< 0.001
Triglyceride (mg/dL)	158.00 [111.50, 213.00]	87.00 [63.00, 115.00]	< 0.001	177.19 [150.44, 242.11]	95.61 [71.05,125.44]	< 0.001
HDL–C (mg/dL)	46.00 [39.00, 54.00]	59.00 [50.00, 69.00]	< 0.001	45.00 [38.08, 51.35]	58.46 [50.00, 68.46]	< 0.001
LDL–C (mg/dL)	140.00 [116.00, 163.00]	132.00 [110.50, 155.00]	< 0.001	140.77 [119.62, 166.92]	126.15 [103.08, 150.00]	< 0.001
Systolic BP (mmHg)	132.00 [119.50, 141.50]	115.50 [105.50, 126.00]	< 0.001	135.46 [119.92,151.00]	121.39 [105.40,137.38]	< 0.001
Diastolic BP (mmHg)	78.50 [71.12, 85.50]	72.50 [67.00, 79.00]	< 0.001	83.19 [73.82,92.56]	74.87 [65.61,84.12]	< 0.001
Fasting glucose (mg/dL)	104.00 [98.00, 115.00]	91.00 [86.00, 95.00]	< 0.001	104.51 [95.50, 113.51]	93.69 [88.29, 99.10]	< 0.001
HbA_1c_ (%)	5.70 [5.40, 6.00]	5.40 [5.10, 5.60]	< 0.001	5.40 [5.10,5.70]	5.10 [4.80,5.40]	< 0.001
Alcohol intake (g/day)	5.71 [0.00, 21.94]	5.71 [0.00, 20.00]	0.723	4.23 [0.76,11.32]	3.97 [1.31,10.04]	0.838
Smoking (%)			< 0.001			< 0.001
Nonsmoker	403 (42.1)	783 (42.2)		77 (36.5)	340 (43.8)	
Former smoker	432 (45.1)	698 (37.6)		93 (44.1)	261 (33.6)	
Current smoker	123 (12.8)	373 (20.1)		40 (19.0)	176 (22.7)	
Physically active (%)	455 (47.5)	1089 (58.7)	< 0.001	142 (67.3)	585 (75.3)	0.011
Intake of fibrates (%)	10 (1.0)	1 (0.1)	< 0.001	^b^NA	^b^NA	
Antihypertensive (%)	545 (56.8)	300 (16.2)	< 0.001	103 (48.8)	172 (22.1)	< 0.001
Antidiabetes (%)	137 (14.3)	12 (0.6)	< 0.001	^b^NA	^b^NA	

^a^Data were median [25th–75th percentile] for quantitative variables and number (percentage) for categorical variables. Wilcox–tests for non-normal distribution continues variables, and chi-square tests with continuity correction for categorical variables

^b^Lacking information in the study

The SHIP-TREND-0 study included 988 participants, comprising 211 individuals diagnosed with MetS and 777 non-MetS participants. Among the MetS individuals, just over half (56%) were male. Consistent with the KORA F4 study, a closer inspection of the table shows significant differences between MetS and non-MetS groups in all included variables except for alcohol intake (Table 1, Fig. 1).

### Identified and replicated 56 MetS-associated metabolites

**Fig. 2:**
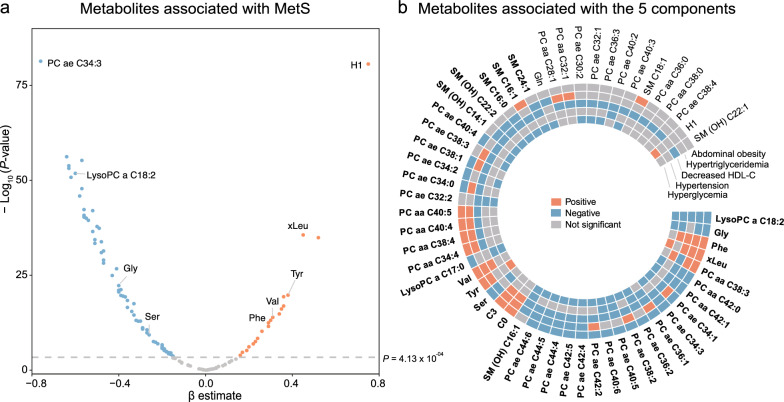
The metabolites associated with MetS and its five components. **a** Volcano plot of regression results of 121 metabolites. The horizontal dashed lines represent the Bonferroni cut-off value. Orange dots: positively associated, blue dots: negatively associated, gray dots: not significant. **b** Circle plot of the association between 56 replicated MetS-associated metabolites and 5 components of MetS. Metabolites with bold font indicate significant association with three or more components

Of the 121 utilized metabolites, we identified 89 metabolites that were significantly (*P*-value < 0.05/121 = 4.13E-04) associated with MetS based on the multiple linear regression models when comparing 959 participants with MetS to 1856 individuals without MetS in the KORA F4 study (Fig. 2a and Table 2). We further performed replications of the 89 MetS-associated metabolites in the SHIP-TREND-0 study. Since metabolite C8:1 was not measured in the SHIP-TREND-0 study due to the different analytical measurement kits, we used the remaining 88 metabolites for replication. Among these, 85 metabolites had the same effect direction in both studies, and 56 remained significant after correction for multiple testing, thus considered successfully replicated (*P*-value < 0.05/88 = 5.68E-04, Table 2). For example, acyl-alkyl PC with 34 carbons and three double-bonds (PC ae C34:3) demonstrated a negative association with MetS in KORA F4 (ß = − 0.76, *P* = 4.06E-82) and SHIP-TREND-0 (ß = − 0.73, *P* = 1.08E-20). Among the successfully replicated metabolites, 13 metabolites (4 amino acids (Val, xLeu (Leu/Ile), Phe and Tyr), 1 monosaccharide (sum of hexoses, H1), 2 acylcarnitines, and 6 PCs) were positively associated with MetS, and 43 metabolites (3 amino acids (Gly, Ser, and Gln), 30 PCs, 2 LPCs, 8 sphingomyelins) were negatively associated with MetS (Table 2).

**Table 2:** MetS-associated metabolites identified in KORA F4, and replicated in SHIP-TREND-0

Metabolites	KORA F4		SHIP-TREND-0	
	β (95% CI)	*P-*value	β (95% CI)	*P*-value
C0	0.20 (0.12, 0.28)	**6.95E-07**	0.32 (0.18, 0.46)	**1.22E-05**
C10	− 0.18 (− 0.26, − 0.10)	**1.43E-05**	− 0.10 (− 0.26, 0.05)	1.96E-01
C12	− 0.23 (− 0.31, − 0.14)	**4.40E-08**	− 0.16 (− 0.31, − 0.00)	4.47E-02
C14:1	− 0.20 (− 0.28, − 0.12)	**1.07E-06**	− 0.20 (− 0.36, − 0.05)	9.85E-03
C14:2	− 0.16 (− 0.24, − 0.08)	**7.22E-05**	− 0.23 (− 0.39, − 0.07)	3.89E-03
C18	− 0.17 (− 0.25, − 0.09)	**2.90E-05**	− 0.19 (− 0.34, − 0.03)	1.75E-02
C3	0.30 (0.22, 0.37)	**7.20E-14**	0.30 (0.15, 0.45)	**8.91E-05**
C5	0.26 (0.18, 0.33)	**5.60E-11**	0.14 (− 0.01, 0.29)	7.53E-02
C8:1	0.19 (0.11, 0.27)	**6.45E-06**	NA	NA
Gln	− 0.34 (− 0.43, − 0.26)	**3.36E-16**	− 0.29 (− 0.45, − 0.14)	**2.76E-04**
Gly	− 0.40 (− 0.48, − 0.32)	**5.12E-23**	− 0.35 (− 0.51, − 0.20)	**9.06E-06**
His	− 0.20 (− 0.28, − 0.12)	**7.69E-07**	0.03 (− 0.13, 0.19)	7.04E-01
Phe	0.29 (0.21, 0.37)	**2.90E-12**	0.32 (0.17, 0.48)	**4.84E-05**
Pro	0.29 (0.21, 0.37)	**3.44E-13**	0.17 (0.02, 0.32)	2.58E-02
Ser	− 0.26 (− 0.34, − 0.18)	**4.93E-10**	− 0.37 (− 0.53, − 0.21)	**3.87E-06**
Tyr	0.38 (0.30, 0.46)	**1.66E-20**	0.41 (0.26, 0.56)	**9.45E-08**
Val	0.31 (0.23, 0.39)	**1.20E-14**	0.45 (0.30, 0.60)	**2.55E-09**
xLeu	0.45 (0.38, 0.52)	**2.33E-36**	0.38 (0.24, 0.52)	**5.90E-08**
lysoPC a C17:0	− 0.29 (− 0.37, − 0.20)	**1.34E-11**	− 0.34 (− 0.49, − 0.18)	**2.01E-05**
lysoPC a C18:0	− 0.17 (− 0.26, − 0.09)	**4.72E-05**	0.26 (0.11, 0.42)	9.33E-04
lysoPC a C18:1	− 0.47 (− 0.55, − 0.39)	**3.91E-32**	− 0.17 (− 0.32, − 0.01)	3.22E-02
lysoPC a C18:2	− 0.60 (− 0.67, − 0.52)	**1.42E-52**	− 0.41 (− 0.56, − 0.27)	**3.54E-08**
lysoPC a C20:4	− 0.19 (− 0.27, − 0.11)	**2.47E-06**	0.23 (0.09, 0.38)	1.84E-03
lysoPC a C28:1	− 0.20 (− 0.28, − 0.12)	**1.84E-06**	− 0.25 (− 0.41, − 0.10)	1.07E-03
PC aa C28:1	− 0.15 (− 0.23, − 0.07)	**1.88E-04**	− 0.32 (− 0.47, − 0.18)	**1.07E-05**
PC aa C32:1	0.36 (0.29, 0.44)	**4.47E-20**	0.38 (0.23, 0.53)	**8.58E-07**
PC aa C32:3	− 0.20 (− 0.28, − 0.13)	**2.07E-07**	− 0.24 (− 0.38, − 0.09)	1.32E-03
PC aa C34:1	0.17 (0.09, 0.25)	**1.98E-05**	0.11 (− 0.05, 0.26)	1.76E-01
PC aa C34:4	0.24 (0.16, 0.32)	**4.20E-09**	0.29 (0.13, 0.44)	**2.80E-04**
PC aa C36:0	− 0.33 (− 0.41, − 0.25)	**3.02E-15**	− 0.28 (− 0.44, − 0.13)	**2.46E-04**
PC aa C36:1	0.16 (0.08, 0.24)	**1.23E-04**	0.09 (− 0.07, 0.24)	2.74E-01
PC aa C36:3	0.22 (0.14, 0.30)	**1.20E-07**	0.20 (0.05, 0.35)	1.09E-02
PC aa C36:4	0.23 (0.15, 0.31)	**2.83E-08**	0.22 (0.06, 0.37)	5.82E-03
PC aa C38:0	− 0.36 (− 0.44, − 0.27)	**2.95E-17**	− 0.30 (− 0.45, − 0.15)	**1.05E-04**
PC aa C38:1	− 0.29 (− 0.37, − 0.21)	**7.49E-12**	− 0.24 (− 0.40, − 0.08)	3.54E-03
PC aa C38:3	0.52 (0.44, 0.60)	**1.22E-35**	0.42 (0.27, 0.58)	**4.78E-08**
PC aa C38:4	0.35 (0.27, 0.43)	**7.48E-17**	0.30 (0.15, 0.46)	**1.11E-04**
PC aa C40:2	− 0.22 (− 0.31, − 0.14)	**1.10E-07**	− 0.17 (− 0.33, − 0.02)	3.10E-02
PC aa C40:3	− 0.18 (− 0.27, − 0.10)	**1.19E-05**	− 0.17 (− 0.32, − 0.01)	3.62E-02
PC aa C40:4	0.36 (0.28, 0.44)	**1.23E-17**	0.37 (0.21, 0.52)	**4.80E-06**
PC aa C40:5	0.34 (0.25, 0.42)	**1.52E-15**	0.39 (0.24, 0.55)	**7.36E-07**
PC aa C42:0	− 0.56 (− 0.64, − 0.48)	**4.89E-43**	− 0.34 (− 0.50, − 0.19)	**1.61E-05**
PC aa C42:1	− 0.51 (− 0.59, − 0.42)	**3.86E-34**	− 0.31 (− 0.47, − 0.16)	**9.35E-05**
PC aa C42:2	− 0.40 (− 0.48, − 0.32)	**2.47E-21**	− 0.18 (− 0.34, − 0.02)	2.42E-02
PC aa C42:4	− 0.19 (− 0.27, − 0.11)	**5.78E-06**	− 0.05 (− 0.21, 0.11)	5.46E-01
PC ae C30:2	− 0.17 (− 0.25, − 0.09)	**2.37E-05**	− 0.44 (− 0.58, − 0.30)	**8.61E-10**
PC ae C32:1	− 0.52 (− 0.60, − 0.44)	**2.95E-37**	− 0.62 (− 0.77, − 0.47)	**1.55E-15**
PC ae C32:2	− 0.50 (− 0.57, − 0.42)	**1.40E-38**	− 0.50 (− 0.65, − 0.35)	**8.30E-11**
PC ae C34:0	− 0.27 (− 0.35, − 0.19)	**2.35E-11**	− 0.43 (− 0.58, − 0.29)	**1.37E-08**
PC ae C34:1	− 0.37 (− 0.45, − 0.29)	**4.86E-20**	− 0.53 (− 0.68, − 0.39)	**1.44E-12**
PC ae C34:2	− 0.64 (− 0.71, − 0.56)	**5.85E-57**	− 0.57 (− 0.72, − 0.42)	**1.80E-13**
PC ae C34:3	− 0.76 (− 0.84, − 0.69)	**4.06E-82**	− 0.73 (− 0.88, − 0.58)	**1.08E-20**
PC ae C36:1	− 0.30 (− 0.38, − 0.22)	**1.19E-13**	− 0.52 (− 0.67, − 0.37)	**4.68E-12**
PC ae C36:2	− 0.57 (− 0.64, − 0.49)	**1.49E-48**	− 0.64 (− 0.78, − 0.49)	**1.05E-17**
PC ae C36:3	− 0.51 (− 0.58, − 0.43)	**4.17E-35**	− 0.42 (− 0.57, − 0.26)	**1.08E-07**
PC ae C36:5	− 0.29 (− 0.37, − 0.20)	**1.43E-11**	− 0.22 (− 0.38, − 0.07)	5.15E-03
PC ae C38:0	− 0.19 (− 0.27, − 0.10)	**6.68E-06**	− 0.12 (− 0.27, 0.03)	1.11E-01
PC ae C38:1	− 0.19 (− 0.28, − 0.11)	**4.87E-06**	− 0.31 (− 0.47, − 0.16)	**1.04E-04**
PC ae C38:2	− 0.43 (− 0.51, − 0.35)	**1.15E-25**	− 0.35 (− 0.50, − 0.19)	**9.26E-06**
PC ae C38:3	− 0.20 (− 0.28, − 0.12)	**7.25E-07**	− 0.30 (− 0.45, − 0.15)	**6.23E-05**
PC ae C38:4	− 0.31 (− 0.39, − 0.23)	**1.25E-13**	− 0.33 (− 0.49, − 0.18)	**3.18E-05**
PC ae C38:5	− 0.29 (− 0.38, − 0.21)	**6.77E-12**	− 0.20 (− 0.36, − 0.05)	1.18E-02
PC ae C38:6	− 0.32 (− 0.40, − 0.23)	**7.49E-14**	− 0.21 (− 0.37, − 0.06)	5.92E-03
PC ae C40:1	− 0.27 (− 0.35, − 0.19)	**1.45E-10**	− 0.21 (− 0.36, − 0.05)	1.06E-02
PC ae C40:2	− 0.39 (− 0.47, − 0.31)	**6.04E-22**	− 0.40 (− 0.55, − 0.25)	**1.02E-07**
PC ae C40:3	− 0.48 (− 0.56, − 0.41)	**1.47E-34**	− 0.31 (− 0.46, − 0.15)	**1.04E-04**
PC ae C40:4	− 0.47 (− 0.55, − 0.39)	**9.84E-30**	− 0.38 (− 0.53, − 0.22)	**1.86E-06**
PC ae C40:5	− 0.56 (− 0.64, − 0.48)	**4.55E-41**	− 0.35 (− 0.51, − 0.20)	**9.60E-06**
PC ae C40:6	− 0.48 (− 0.56, − 0.40)	**1.06E-31**	− 0.43 (− 0.57, − 0.28)	**1.75E-08**
PC ae C42:2	− 0.39 (− 0.47, − 0.31)	**2.04E-20**	− 0.29 (− 0.45, − 0.14)	**1.82E-04**
PC ae C42:3	− 0.55 (− 0.62, − 0.47)	**8.88E-41**	− 0.22 (− 0.37, − 0.06)	6.60E-03
PC ae C42:4	− 0.63 (− 0.70, − 0.55)	**6.67E-54**	− 0.47 (− 0.62, − 0.31)	**6.23E-09**
PC ae C42:5	− 0.58 (− 0.66, − 0.51)	**1.32E-46**	− 0.58 (− 0.73, − 0.43)	**2.33E-13**
PC ae C44:3	− 0.40 (− 0.49, − 0.32)	**8.71E-22**	− 0.01 (− 0.17, 0.15)	8.83E-01
PC ae C44:4	− 0.54 (− 0.62, − 0.46)	**3.36E-40**	− 0.28 (− 0.44, − 0.12)	**4.88E-04**
PC ae C44:5	− 0.56 (− 0.64, − 0.48)	**1.58E-41**	− 0.57 (− 0.73, − 0.42)	**5.19E-13**
PC ae C44:6	− 0.62 (− 0.70, − 0.54)	**1.55E-51**	− 0.59 (− 0.74, − 0.43)	**1.83E-13**
H1	0.75 (0.68, 0.83)	**2.18E-81**	0.39 (0.24, 0.55)	**7.73E-07**
SM (OH) C14:1	− 0.52 (− 0.59, − 0.45)	**9.32E-43**	− 0.64 (− 0.77, − 0.50)	**3.10E-19**
SM (OH) C16:1	− 0.49 (− 0.56, − 0.41)	**4.16E-38**	− 0.59 (− 0.73, − 0.45)	**1.51E-16**
SM (OH) C22:1	− 0.36 (− 0.44, − 0.28)	**4.29E-19**	− 0.37 (− 0.52, − 0.22)	**1.15E-06**
SM (OH) C22:2	− 0.57 (− 0.64, − 0.50)	**5.45E-56**	− 0.57 (− 0.70, − 0.43)	**1.72E-15**
SM (OH) C24:1	− 0.38 (− 0.46, − 0.30)	**3.12E-20**	− 0.26 (− 0.41, − 0.10)	1.24E-03
SM C16:0	− 0.63 (− 0.71, − 0.55)	**1.38E-54**	− 0.61 (− 0.76, − 0.46)	**1.23E-15**
SM C16:1	− 0.33 (− 0.40, − 0.25)	**3.01E-18**	− 0.35 (− 0.48, − 0.21)	**8.45E-07**
SM C18:1	− 0.16 (− 0.23, − 0.08)	**2.79E-05**	− 0.26 (− 0.41, − 0.12)	**3.82E-04**
SM C20:2	− 0.41 (− 0.49, − 0.34)	**1.90E-27**	− 0.22 (− 0.37, − 0.08)	3.20E-03
SM C24:0	− 0.24 (− 0.32, − 0.15)	**2.86E-08**	− 0.16 (− 0.32, − 0.00)	4.49E-02
SM C24:1	− 0.47 (− 0.55, − 0.39)	**6.15E-29**	− 0.47 (− 0.62, − 0.31)	**4.20E-09**

Coefficients with 95% confidence interval (CI) and P-values of multiple linear regression adjusted for age, sex, smoking, physical activity, and alcohol intake. Bold P values indicate statistical significance at levels of P-value < 0.05/121 = 4.13E-04 for the KORA F4 study and P-value < 0.05/88 = 5.68E-04 for the SHIP-TREND-0 study, respectively

### Metabolites associated with the five components

**Fig. 3:**
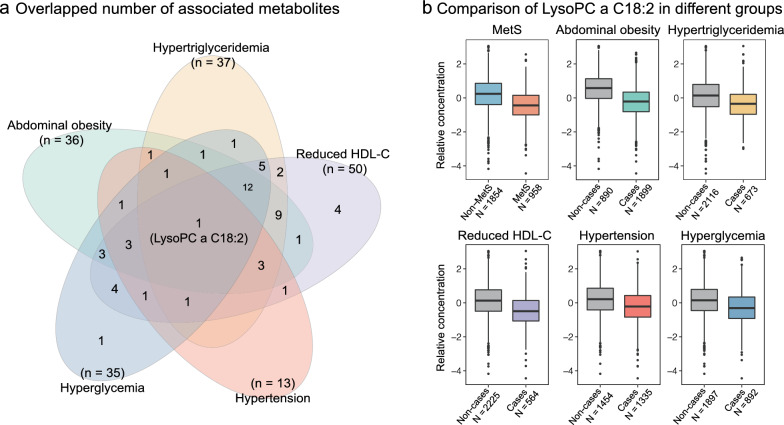
The number of overlapped metabolites among the five components and comparison of the overlapped metabolite. **a** The Venn diagram displays the number of metabolites that were Bonferroni significantly associated with each component. **b** Box plots shows the log-transformed and scaled concentrations of lysoPC a C18:2 in the MetS group, the five components of MetS groups, and the corresponding non-case groups

We further investigated whether and how the identified and replicated 56 MetS-associated metabolites were associated with each of the five components of MetS. Using the multiple logistic regression analyses, we found that abdominal obesity, hypertriglyceridemia, reduced HDL–C, hypertension, and hyperglycemia were significantly (*P*-value < 0.05/56 = 8.93E-04) associated with 36, 37, 50, 13, 35 metabolites, respectively (Figs. 2b, 3a, Additional file 1: Table S2). In addition, lysoPC a C18:2 was the only metabolite significantly negatively associated with all five components: abdominal obesity (odds ratio, OR = 0.57, *P* = 3.96E-23), hypertriglyceridemia (OR = 0.69, *P* = 6.57E-10), low HDL–C (OR = 0.65, *P* = 1.34E-12), hypertension (OR = 0.82, *P* = 2.62E-04), and hyperglycemia (OR = 0.81, *P* = 1.24E-04) (Additional file 1: Table S2). The concentration of lysoPC a C18:2 was lower in individuals with MetS and in those with each of the five components compared to respective controls (Fig. 3b). Furthermore, BCAAs (Val, xLeu (Leu/Ile) and aromatic amino acids (Phe and Tyr) were positively associated with at least three of the five components (Fig. 2b). The concentrations of Val, xLeu, Phe and Tyr were higher in participants with abdominal obesity, hypertriglyceridemia, reduced HDL–C (except for Tyr), hypertension (except for BCAAs and Tyr), and hyperglycemia (except for Val and Phe), when compared to corresponding controls, respectively (Additional file 1: Table S2). Moreover, two acylcarnitines (C0 and C3) showed positive associations with abdominal obesity, hypertriglyceridemia, and reduced HDL–C (Fig. 2b).

### Three created metabolic networks

**Fig. 4:**
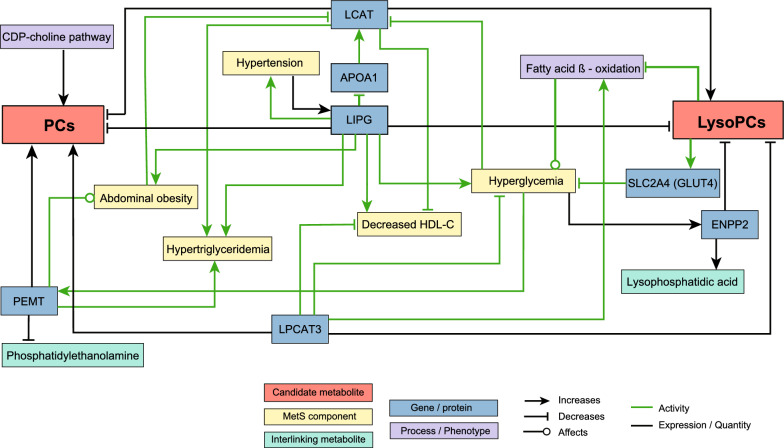
Metabolic network of PCs and LPCs. *LCAT* lecithin cholesterol acyltransferase, *APOA1* apolipoprotein A1, *LIPG* endothelial lipase, *LPCAT3* lysophosphatidylcholine acyltransferase-3, *PEMT* phosphatidylethanolamine N-methyltransferase, *ENPP2* ectonucleotide pyrophosphatase/phosphodiesterase 2, *SLC2A4* solute carrier family 2, facilitated glucose transporter member 4

**Fig. 5:**
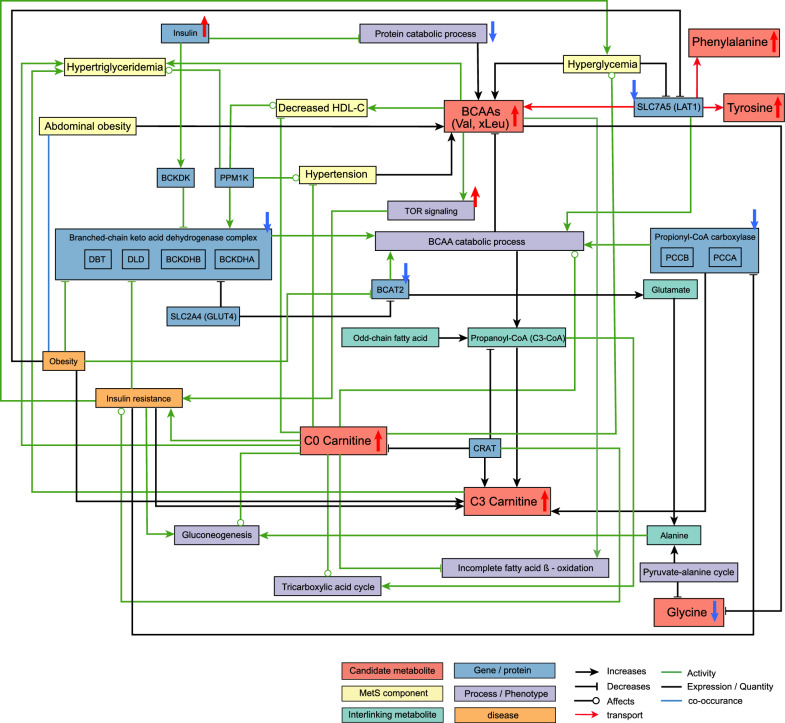
Metabolic network of BCAAs. *BCKDK* branched chain ketoacid dehydrogenase kinase, *BCAT2* Branched Chain Amino Acid Transaminase 2, *SLC7A5* Solute Carrier Family 7 Member 5, *PPM1K* Protein phosphatase 1 K, *SLC2A4* solute carrier family 2, facilitated glucose transporter member 4, *CRAT* carnitine acetyltransferase

**Fig. 6:**
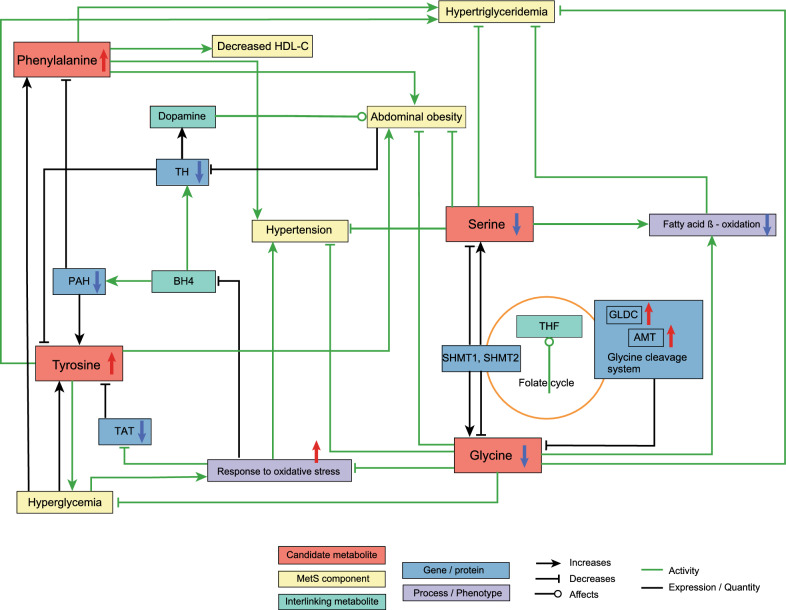
Metabolic network of Ser, Gly, Phe, and Tyr. *TH* tyrosine hydroxylase, *PAH* phenylalanine hydroxylase, *TAT* tyrosine aminotransferase, *BH4* tetrahydrobiopterin, *THF* tetrahydrofolate, *GLDC* glycine decarboxylase, *AMT* aminomethyltransferase, *SHMT* serine hydroxymethyltransferase

We constructed three networks by systematically investigating the identified and replicated MetS-associated metabolites with MetS and its five components based on the CIDeR database. We found that the conversions between PCs and LPCs are mediated by multiple enzymes (e.g., LCAT, lecithin cholesterol acyltransferase; LIPG, endothelial lipase; LPCAT3, lysophosphatidylcholine acyltransferase—3) (Fig. 4 and Additional file 1: Table S3). All of these three enzymes are functionally interacting with at least one of the five components. For example, hypertension regulates LIPG's activity which can hydrolyze the fatty acid from both PCs and LPCs (Fig. 4). Additionally, LIPG’s concentration was reported positively associated with increased abdominal obesity (waist circumference), hypertriglyceridemia, hyperglycemia, and decreased HDL–C (Fig. 4). To further explore underlying molecular mechanisms, we created two networks of amino acids and acylcarnitines (Figs. 5, 6, see "Discussion").

## Discussion

MetS is a complex syndrome derived to capture the interplay of risk factors for cardiometabolic diseases. To better understand the underlying molecular mechanisms, we analyzed more than 3800 study participants in two population-based human cohorts (KORA F4 and SHIP-TREND-0). Of 121 metabolites, 56 were identified and replicated as MetS-associated metabolites and further evaluated with each of the five components representing a criterion for the MetS. The creation of three metabolic networks facilitated the understanding of the interplay between metabolites, proteins, biomedical processes, phenotypes, and MetS components.

Our findings highlight H1 as the metabolite with the most positive association with MetS (Fig. 2a, Table 2), and exclusively associated with the component of hyperglycemia (Fig. 2b, and Additional file 1: Table S2). Based on the targeted metabolomics technology, we were unable to distinguish between different hexoses. Therefore, it is likely that the level of H1 is mainly represented by glucose and thereby directly linked to the definition of hyperglycemia, as expected [16].

We also found that 36 PCs were associated with MetS. Biological membranes are composed primarily of phospholipids, and phospholipids additionally serve as substrates to produce bioactive compounds, and the most abundant phospholipids in mammalian cell membranes are PCs [35]. PCs play an important role in lipoprotein metabolism by regulating the expression of key factors in lipoprotein production [36]. Consistent with our observations, four long-chain acyl-alkyl PCs (PC ae C42:5, PC ae C44:5, PC ae C40:4, and PC ae C44:4) were inversely associated with MetS in a study of the Health Survey of Sao Paulo, which analyzed plasma samples from 130 participants [37]. Another study analyzed morning urine samples and observed a higher concentration of metabolite PC (34:2) in individuals with MetS (N = 30) when compared to controls (N = 20) [22]. However, no significant association between PC aa C34:2 and MetS was found in the serum samples of the KORA F4 study. This discrepancy could be attributed to a multitude of factors, including the limited sample size utilized in the analysis and the potential impact of using urine samples on the specificity of the results.

We observed that two LPCs were negatively associated with MetS, and especially, lysoPC a C18:2 was significantly associated with all five MetS components. There is an increasing awareness of the connection between LPCs and cardiovascular and neurodegenerative disorders [38]. Prior studies have shown that LPC was important for the mechanism connecting saturated fatty acids to insulin resistance [39]. Using the targeted metabolomics approaches, it is shown that lower levels of lysoPC a C18:2 are not only found in adults with impaired glucose tolerance [16], but also in obese children [40], and this lipid is independently associated with decreased risk of T2D [17] as well as myocardial infarction [41]. These findings exhibit consistency with our results. Besides, LIPG has the ability to hydrolyze fatty acids from both PCs and LPCs, and LIPG has a significant association with features of MetS, it may potentially serve as a pro-atherogenic factor in individuals with MetS (Fig. 4) [42]. Furthermore, LIPG has been shown to produce LPC18:2 from PCs [43]. This implies that LIPG has an important role in the pathogenesis of MetS, as it is not only associated with the five MetS components (Fig. 4 and Additional file 1: Table S3), but also with lysoPC a C18:2, which is also associated with all five components in our study. In addition, lysoPC a C18:2 may also be involved in the regulation of lipid metabolism, inflammation, and glucose homeostasis, which are key factors in the development and progression of MetS [16, 17, 41]. However, we are just beginning to understand how PCs and LPCs are metabolically regulated in health and disease. To understand the mechanisms that underlie PC’s and LPC’s potential roles in metabolic diseases, future functional studies will be necessary.

Among the 14 amino acids, we found seven (Val, xLeu, Phe, Tyr, Ser, Gly, and Gln) were significantly associated with MetS. Consistent with previous studies, Ser was reported to be inversely associated with MetS in the Health Survey of Sao Paulo study [37]. Additionally, a previous study of 56 MetS participants found a positive association between MetS and BCAAs and aromatic amino acids, which is in line with our findings [23]. Given that meats and meat products are rich sources of BCAAs and aromatic amino acids, these observations could be a sign of meat consumption [44].

Recently, an untargeted metabolomics approach based on nuclear magnetic resonance technology was used to analyze about 1000 plasma samples of a community-based study in a Chinese population [20]. Of 85 used metabolites, 13 (e.g., d-maltose, d-fucose, and l-ornithine) were selected as candidate biomarkers for MetS and it was concluded that arginine metabolism may play a role in the pathophysiological mechanism of MetS [20]. The observations of d-maltose and d-fucose are consistent with our H1 hexoses observations, however, both arginine (ß = − 0.11, *P* = 1.26E-02) and ornithine (ß = 0.01, *P* = 9.04E-01) were not significantly associated with MetS in the KORA F4 study. 

We observed a positive association of BCAAs not only with MetS, but also with abdominal obesity, hypertriglyceridemia, and reduced HDL–C. The expression of SLC7A5 (solute carrier family 7 member 5, also known as LAT1, the transporter of BCAAs) was reported to be reduced in obese individuals, in addition, several enzymes associated with BCAA catabolism, such as propionyl-coA carboxylase (PCC), branched-chain ketoacid dehydrogenase (BCKD) complex and branched-chain amino acid transaminase 2 (BCAT2) were reported down-regulated (Fig. 5 and Additional file 1: Table S4). Specifically, mitochondrial branched-chain amino acid aminotransferase (BCATm) and BCKD E1α were found reduced in adipose tissues, but not in skeletal muscles [45, 46]. Down-regulated enzymes observed in obese individuals and adipose tissues impair BCAA catabolism, consequently leading to raised BCAA levels in the circulation, as we found in serum samples (Fig. 5 and Additional file 1: Table S4). Moreover, we observed that both carnitines (C0 and C3) were positively associated with MetS, abdominal obesity, hypertriglyceridemia, and reduced HDL–C, respectively. C3 is a product of BCAA catabolism, which is impaired in MetS, thus C3 produced from BCAA should have been reduced in individuals with MetS. Our observation of raised C3 levels may be attributable to alternative causes (e.g. ß-oxidation of fatty acids with odd chain length, or the tricarboxylic acid cycle) (Fig. 5 and Additional file 1: Table S4). Raised BCAA and raised C3 levels have been found in individuals with insulin resistance, and the authors suggested that the BCAA catabolism might be impaired in adipose tissue but increased in other tissues such as skeletal muscle [47]. It remains an area of ongoing controversy and research whether BCAAs are a contributor or the result of insulin resistance [48]. The causal relationship between elevated levels of BCAAs, C0, and C3, and metabolic disturbances remains unclear. The regulation of BCAAs seems to be tissue-dependent and influenced by nutrition and health status, highlighting their potential role in obesity, diabetes, and multifactorial diseases such as MetS [49].

We observed that aromatic amino acids (Phe and Tyr) were positively associated with MetS. Phe is one of the essential amino acids in the human body, while Tyr is a semi-essential amino acid that can be synthesized from Phe. Three enzymes (PAH, phenylalanine hydroxylase, TH, tyrosine hydroxylase, and TAT, tyrosine aminotransferase) are reported to be responsible for the catabolism of these aromatic amino acids (Fig. 6 and Additional file 1: Table S5). PAH catalyzes the hydroxylation of Phe to Tyr [50]. Tyr can be further catabolized by TH to dopamine or by TAT to 4-hydroxyphenylpyruvate [51, 52]. Reduced expression of PAH was found in pigs with MetS [53], TH expression was found lower in individuals with central obesity [54], and lowered expression of TAT in the liver was reported in insulin-resistant mice [51].

We also found that Gly and Ser showed significant negative associations with MetS and its associated conditions, including increased waist circumference, hypertriglyceridemia, hypertension, and hyperglycemia (not for Ser). Gly is the simplest stable proteinogenic amino acid and can be biosynthesized from Ser in the body. Intake of Gly and Ser was reported to boost the rate of fatty acid oxidation and reduce triglyceride production [55, 56]. (Fig. 6). Human serine hydroxymethyltransferases (SHMTs) catalyze the conversion of Ser and Gly and vice versa, a process that is linked to the folate cycle [57, 58]. Additionally, the breakdown of Gly by the glycine cleavage system (GCS) is also associated with the folate cycle [59] (Fig. 6). Interestingly, the expression of two enzymes in the GCS, glycine decarboxylase (GLDC), and aminomethyltransferase (AMT), were found to be increased in obese rats [60] (shown with red arrows in Fig. 6). Folate treatment in clinical trials has shown a potential to improve insulin resistance and endothelial dysfunction in MetS patients [61]. Therefore, the increased catabolism of Gly may contribute to the reduction in Gly levels. Both Ser and Gly play important roles in maintaining cellular oxidative homeostasis [62]. Studies have shown that oral supplementation with Gly protects against oxidative damage in individuals with MetS [63]. On the other hand, reductions in Gly and Ser may lead to increased levels of reactive oxygen species (ROS) [64, 65], which could further inhibit the catabolism of Tyr and Phe by limiting the availability of the cofactor tetrahydrobiopterin (BH4) required by TH and PAH [66]. Moreover, TAT activity can be inhibited under oxidative stress conditions [67]. Consequently, the catabolism of Tyr and Phe could also be further reduced by higher ROS concentrations due to low Gly and Ser levels. In light of these findings, it seems that the raised concentrations of Tyr and Phe, and lowered levels of Gly and Ser observed in our study, are a consequence of the down-regulated expression and activity of PAH, TH, and TAT, and up-regulated GLDC and AMT expression (Fig. 6 and Additional file 1: Table S5). Moreover, raised levels of Gly and Ser decreased hypertension, abdominal obesity, and hypertriglyceridemia. This may indicate a protective effect of these two metabolites against MetS (Fig. 6 and Additional file 1: Table S5).

A limitation of our study is that one metabolite identified in the discovery study could not be replicated in SHIP-TREND-0 due to the use of different analytical kits. In addition, contrary to the KORA F4 study, the metabolomics quantification in the SHIP-TREND-0 study was performed only on a subgroup of participants who did not report having diabetes. Thus, the study was designed to include a relatively healthy cohort of participants, with non-diabetic individuals and, consequently, no diabetes-related diseases. Despite these structural differences in the study design, the study included participants with prediabetes, along with a sufficient number of metabolites. Moreover, the successful replication of the majority of the results indicated that these results are very robust and generalizable. An additional limitation of the study is that metabolites such as PCs and LPCs represent a group of possible metabolites rather than a single molecule. For instance, the metabolite lysoPC a C18:2 is known for its carbon chain length of the fatty acid and number of double bonds, but the position of the double bonds remains undefined. Therefore, the fatty acid in this LPC might be linoleic acid (C18:2 9Z, 12Z) or conjugated linoleic acid (C18:2 9E, 11E or C18:2 10E, 12Z) with different positions of the double bonds. For the PCs, the range of possible molecules is even wider, since we know the sum of the chain lengths and the sum of the double bonds of the two fatty acids in PC, but not which fatty acid is found at the sn1 or sn2 position. The level of the PCs or LPCs is determined by biosynthesis or catabolism, and the enzymes involved in these processes often exhibit a preference for specific fatty acids at specific positions. However, the substrate spectrum for all the enzymes is not yet fully known. Consequently, we were unable to construct networks for specific PCs or LPCs in our metabolic network analysis.

## Conclusion

MetS is a complex and multifaceted syndrome, characterized by overlapping and interacting mechanisms that are not identical across individual phenotypes. A notable finding from our study is the identification of lysoPC a C18:2, a metabolite negatively associated not only with MetS but also with its five components. This metabolite might have the potential to be a key molecule in the prevention and treatment of MetS. Consequently, a greater and deeper comprehension of the lysoPC a C18:2 metabolic process, and its effect on the development of MetS is required. Additionally, the biological processes and enzymes involved in the network analysis of specific replicated MetS-associated metabolites can also be explored for preventive and therapeutic purposes.

## Supplementary Information

**Additional file 1: Table S1.** Metabolite quantification and normalization in KORA F4. The table presents metabolite abbreviations, analyte classes, and biochemical names of 163 metabolites in columns first to third. Detecting rate of each metabolite in 3,061 KORA F4 individuals is shown in the fourth column. Column fifth displays the median coefficient of variation of 114 quality control samples. The sixth column displays the percentage of 3061 KORA F4 study individuals with values at or above the limit of detection. The final column displays each metabolite’s used/excluded status. **Table S2.** Logistic regression results of MetS-associated metabolites with each of the 5 components in the KORA study. Odds ratioswith 95% confidence interval. Significant P-value < 0.05/56 = 8.93E-04 are shown in bold. **Table S3.** Metabolic network of PCs and LPCs. **Table S4.** Metabolic network of BCAAs. **Table S5.** Metabolic network of Ser, Gly, Phe, and Tyr.

## Data Availability

Both KORA and SHIP data sets are not publicly available because of data protection agreements but can be provided upon request through the KORA-PASST (Project application self-service tool, https://helmholtz-muenchen.managed-otrs.com/external) and the SHIP transfer office (https://www.fvcm.med.uni--greifswald.de/cm_antrag/index.php).

## Abbreviations

AMT: Aminomethyltransferase
BCAAs: Branch-chain amino acids
BCAT2: Branched chain amino acid transaminase 2
BCATm: Mitochondrial branched chain amino acid aminotransferase
BCKD: Branched-chain ketoacid dehydrogenase
BH4: Tetrahydrobiopterin
CV: Coefficient of variance
CVD: Cardiovascular disease
F4: The first follow-up study of the KORA survey number 4
GLDC: Glycine decarboxylase
GCS: Glycine cleavage system
HDL–C: High density lipoprotein cholesterol
KORA: Cooperative Health Research in the Region of Augsburg
LCAT: Lecithin cholesterol acyltransferase
LIPG: Endothelial lipase
LOD: Limits of detection
LPC: Lysophosphatidylcholine
LPCAT3: Lysophosphatidylcholine acyltransferase-3
MetS: Metabolic syndrome
PAH: Phenylalanine hydroxylase
PC: Phosphatidylcholines
PCC: Propionyl-coA carboxylase
QC: Quality control
ROS: Reactive oxygen species
RSD: Relative standard deviations
SARS-CoV-2: Severe acute respiratory syndrome coronavirus 2
SHIP: The Health Study of Pomerania
SHMT: Serine hydroxymethyltransferase
SLC7A5: Solute Carrier Family 7 Member 5
TAT: Tyrosine aminotransferase
TH: Tyrosine hydroxylase
